# The Experience of Depression during the Careers of Elite Male Athletes

**DOI:** 10.3389/fpsyg.2016.01069

**Published:** 2016-07-18

**Authors:** Steve Doherty, Barbara Hannigan, Mark J. Campbell

**Affiliations:** ^1^School of Psychology, Trinity College DublinDublin, Ireland; ^2^Health Research Institute – Department of Physical Education and Sport Sciences, University of LimerickLimerick, Ireland

**Keywords:** male, depression, elite sport culture, Identity, masculinity

## Abstract

The topic of depression during the career of elite male athletes has been the subject of much public interest and attention in recent years. Despite numerous debates and personal disclosures within the media, there is a dearth of published research directly exploring the phenomenon. This study sought to explore how elite male athletes experience depression during their sporting careers. Eight former/current elite male athletes who had previously publically self-identified as having experienced depression while participating in sport were recruited for this study. A qualitative methodology was employed and each participant was interviewed using semi-structured interviews. Data analysis which was conducted using descriptive and interpretive thematic analysis uncovered three domains: (1) The emergence of depression, (2) The manifestation of symptoms of depression, and (3) Adaptive and Maladaptive proceesses of recovery. Findings from the current study reveal the nature of how male athletes experience, express, and respond to depression during their careers. Additionally, this is influenced by a myriad of factors embedded in the masculine elite sport environment. Implications are discussed particularly in relation to atypical expressions of depression not necessarily reflected on or in standard diagnostic criteria. Future research is encouraged to examine in depth moderating factors (e.g., athletic sense of identity and masculine elite sport environments) for the relationship between depression and participation in elite sport.

## Introduction

Within the Diagnostic statistical manual of mental disorders (DSM-V; American Psychiatric Association [APA], 2013), numerous disorders fall under the category of depression. While the specific criteria for these disorders differ, individuals diagnosed with depression are generally observed to experience a reduction in functioning (e.g., occupational) while presenting with a range of persistent symptomology. Such symptoms include; experiences of low mood, sadness, decreased energy and motivation, feelings of worthlessness or guilt, difficulties in concentration, changes in appetite, problems sleeping and recurrent thoughts of death or suicidal ideation over at least a 2-week period (American Psychiatric Association [APA], 2013).

Recently, there has been an upsurge of interest in qualitative explorations of male depression (Martin et al., 2013). While the components of depression vary throughout these studies, they tend to describe overlapping, cyclical, suppressive, avoidant, and externalizing symptomatology or attempts to mask depression. Participants in these studies have consistently described experiences related to; increased interpersonal withdrawal, substance abuse, increased frequency of interpersonal conflict, self-destruction, an over investment in work, avoidance of help seeking and an escalation in anger outbursts (Brownhill et al., 2005; Chuick et al., 2009; Oliffe et al., 2010). Researchers have hypothesized that learned typical gender norms such as dominance, emotional control, avoidance of femininity, risk taking, pursuit of status and winning, primacy of work and extreme self-reliance encourage the manifestation of these atypical symptoms or “depression equivalents” (Cochran and Rabinowitz, 2000; Mahalik et al., 2003; Brownhill et al., 2005). While the processes which underlie and are assumed to be involved in the experience of more masculine forms of depression are not directly supported by empirical research (Addis, 2008), indirect evidence broadly supports the theory that traditional masculine cultures shape how men experience, express and respond to depression (Addis and Cohane, 2005; Cochran, 2005). It is on this basis that scholars often question the prevalence rates for depression in the male population. The DSM-V (American Psychiatric Association [APA], 2013) criteria for the disorder which is regularly employed within epidemiological research studies has been hypothesized to represent a more feminine congruent coping process while not reflecting the manifestation of more masculine expressions of depression (Kilmartin, 2005). This body of research has fuelled subsequent studies that have attempted to establish more efficient methods of assessing manifestations of depression in men (Martin et al., 2013).

### Athlete Mental Health

The topic of mental health in sport has not received that much attention within academic literature. Reardon and Factor (2010) claim that our tendency to idealize elite athletes has led the general public and some within the healthcare profession to assume a low prevalence of mental health issues in sport. Problems related to recognizing psychological difficulties within an elite sport culture have also been hypothesized. For example, a review carried out by Thompson and Sherman (1999) discussed how the specific manifestation of Anorexia Nervosa such as overtraining or denying discomfort could be confused for what they termed ‘good athlete characteristics.’ Furthermore, as empirical research reports that athletes have a negative perception of help-seeking (Steinfeldt and Steinfeldt, 2012) and often accept pain while minimizing displays of weakness (Sinden, 2010), it could be inferred that they may be less likely to willingly present to mental health professionals for support related to psychological distress during their careers. Numerous high profile individuals retrospectively reporting episodes of depression during their sporting careers has fuelled a recent surge of public and media interest (Trescothick, 2008; Kirwan and Thomson, 2010). While epidemiological research on the prevalence of depression and various forms of psychopathology in the male or female elite sport context does exist, it is limited and mired with inconsistent findings. For example, consider the large contrast in the following two studies; Resch and Haasz (2009) suggested that prevalence rates for depression were 37.5% amongst the athletes recruited to participate in their study, while Schaal et al. (2011) observed that only 1% of the athletes within their study suffered from Major Depressive Disorder (MDD). It is worth noting that Resch and Haasz (2009) administered surveys to athletes and focused their study primarily on eating disorders, while Schaal et al. (2011) recruited both high level and junior level elite athletes and derived their results from nationwide data that were obtained from the athletes yearly psychological evaluations. While the topic continues to gain traction within media circles, based on the current available evidence in an elite sport context, it remains challenging, at present, to quantify the precise extent and nature of athlete’s problems with depression during their careers.

### The Costs Associated with Competing in Elite Sport and Their Link to Mental Health

A plethora of studies have presented the positive and protective factors associated with engaging in sport and exercise (Buckworth and Dishman, 2002; Rethorst et al., 2009). Since Miller and Kerr’s (2002) review article, there has been a substantial increase in research seeking to understand the person behind the athlete (Warriner and Lavallee, 2008; Carless and Douglas, 2012, 2013).

Numerous researchers have commented on the concerning phenomenon that while athletes strive to achieve excellence within the elite sport environment their identity often becomes completely foreclosed or constructed around their ability to perform in their athletic career (Warriner and Lavallee, 2008; Carless and Douglas, 2009). In addition to the ‘performance narrative’ which some scholars observe as the dominant message that athletes internalize within their day to day lives (Douglas and Carless, 2006), studies have also discussed how athletes are exposed to values that serve to reinforce qualities (competition, aggression, and toughness) which are often associated with traditional conceptualisations of masculinity (Steinfeldt and Steinfeldt, 2012). Various papers have added to our understanding of masculinity and the possible negative consequences associated with constructions of strength and toughness in sport (Young et al., 1994; Wacquant, 2001; Sinden, 2010). Elite rowers retrospectively reported actively suppressing emotions to avoid appearing mentally weak, negative, or irrational while suffering from health problems during training (Sinden, 2010). Further papers which have covered topics such as organizational stress (Fletcher et al., 2012), extrinsic motivation (Lemyre et al., 2007), burnout (Cresswell and Eklund, 2007), attitudes to help seeking (Steinfeldt and Steinfeldt, 2012), risk taking (Schnell et al., 2014), and adversity (Howells and Fletcher, 2015) have prompted numerous researchers to speculate that athletes are vulnerable to developing mental health issues (Reardon and Factor, 2010; Hughes and Leavey, 2012).

While a number of studies have indirectly mentioned depressive mood within their discussions (e.g., Brewer et al., 1993; Carless and Douglas, 2009), from the outset these studies have not specifically focused on the construct of depression. Furthermore, while there are an increasing number of review articles on the subject of mental health and depression in elite sport (Reardon and Factor, 2010; Hughes and Leavey, 2012) there currently is a dearth of research directly exploring the phenomenon.

### Rationale for Current Study

The last decade has seen a growing acceptance of qualitative methods within the sport psychology domain (Biddle et al., 2001) and the authors of this paper argue that there is also a need for employing this paradigm when attempting to understand the phenomenon of depression within the male athletic population. There is a general consensus within sport psychology literature that elite sport has unique challenges, stresses, and constraints (Schaal et al., 2011). While reflecting on the established necessity to understand depression in the context of the culture in which an individual resides (Cochran and Rabinowitz, 2000), it is imperative that researchers seek to explore the subjective experiences of those living within the environment of elite male sport. This seems particularly important considering the recent suggestion that practitioners have begun to accept that the psychological care of athletes is being delivered without a full understanding of the diagnostic and therapeutic issues unique to this population (Reardon and Factor, 2010). In many ways it would seem prudent to embrace viewpoints from previous commentators who encouraged researchers to move away from a foreclosed focus on measurement and simply ‘ask men’ and in this case ‘elite male athletes’ about their experiences (Cochran and Rabinowitz, 2000).

To summarize, the present paper utilizes qualitative methods to elucidate the meaning and nature of depression within the context of an elite male athletic career. With the exception of Jones (2010) who asked female non-elite athletes about depression, this topic has received little attention within academic literature. This paper represents the first published study to explore elite male athlete’s experiences of depression during their sporting careers.

## Materials and Methods

### Design

A qualitative methodology was utilized and semi structured interviews were employed as the method of data collection (Smith, 2007).

### Development of Semi-structured Interview

An interview schedule which was developed by the research team and informed by their clinical knowledge and the contemporaneous research literature in the area (e.g., Chuick et al., 2009) was divided into three stages. Within the first stage, attention was directed toward how the athletes *understood* the initial development and experience of depression during their careers. The second stage focused on how elite male athletes *expressed* their depression during their careers, while the third and final stage of the interview focused on how elite male athletes *coped* with their encounter(s) with depression during their careers. Evocative wording, prompts and the empathic communication skills of the researcher were used in the interview with a view to encouraging the participants to reflect deeply on their experiences. Prior to beginning the research interviews, a pilot interview was conducted with a college athlete who had previously experienced depression. Based on this pilot study, the draft interview schedule was modified to incorporate the experience of the interview, and feedback from the pilot interviewee.

### Recruitment

Purposeful sampling which has been previously employed in qualitative explorations of various athletes’ experiences (Kirby et al., 2011) was employed during the recruitment stage. Through broad internet searches, 43 males who had publically disclosed having encountered depression during their sporting careers were identified as potential participants. Contact was made with prospective participants through an ethically approved invitation sent via email or through private messages on social media sites in the public domain. Eight out of the 15 who responded to the invitation, agreed to participate in the study.

### Participants

Eight Caucasian male current/former elite athletes from seven different sports who had previously publically self-disclosed having had depression during their sporting careers participated in this study. A recent paper by Swann et al. (2015) critiqued the use of the term ‘elite athlete’ within sport psychology research and suggested that the term varied on a continuum of validity. The authors further translated their findings into a taxonomy for classifying expert samples within future studies and developed an equation to position athlete’s levels of expertise into four categories; semi- elite; competitive elite, successful elite, and world class elite. This model was employed within this paper in an attempt to illustrate participant’s levels of experience and success within their respective sport. As per the Swann et al. (2015) classifications, *semi-elite athletes* are those whose highest level of participation is below the top standard possible in their sport; *Competitive-elite athletes* regularly compete at the highest level in their sport but have not had any success at that level; *Successful-elite athletes* not only compete at the highest level, but have experienced some (infrequent) success at that standard (e.g., winning an event or a medal); *World-class elite athletes* experience sustained success at the highest level, with six repeated wins over a prolonged period of time (e.g., winning gold medals in consecutive Olympics, or major competitive victories over a number of seasons). While anonymity could not be guaranteed due to the public nature of the athletes stories, all efforts were made to anonymize data throughout each stage of the research project. To illustrate how participants met criteria for this project and to provide some context to the analysis process, some pertinent demographic information is provided in **Table 1.**

**Table 1:** Participant demographic information.

Participant (P)	Age	Nationality	Format	Elite level	Diagnosis of depression^∗^	Treatment^†^
1	22	British/Irish	Team	Competitive elite	Yes	Yes
2	29	North America	Individual	Competitive elite	Yes	Yes
3	32	North America	Team	Successful elite	No	No
4	58	British/Irish	Team	World class elite	Yes	Yes
5	45	North America	Individual	Successful elite	Yes	Yes
6	35	British/Irish	Team	Competitive elite	Yes	Yes
7	37	Australia/Oceania	Team	Successful elite	Yes	Yes
8	65	North America	Individual	Successful elite	Yes	Yes


*^∗^Diagnosis- The ‘diagnosis of depression’ column above, indicates whether or not the athlete was formally diagnosed with depression by a mental health professional such as a GP, Psychiatrist, or Psychologist.*

*^†^The treatment column above indicates whether the athlete had ever attended for psychological therapy, such as cognitive behavior therapy or psychodynamic therapy, or similar clinical intervention and or were prescribed medication such as anti-depressants or a similar psychopharmacological treatment indicted for treating depression.*

### Data Collection

As five of the participants recruited in the study were residents in North America and Australia/Oceania these interviews took place on Skype. The remaining three interviews took place in various private locations throughout Ireland and the UK. Data collection occurred between July 2013 and March 2014. Each interview was audio recorded and lasted between 65 and 90 min with a mean length of 79 min and standard deviation of 14.5.

### Data Analysis

Descriptive and Interpretative Analysis (Elliott and Timulak, 2005) was employed to analyze the experiences of depression during elite sport careers. The steps followed during data analysis are outlined below;

(i) Interviews were transcribed verbatim and read and re-read by the principal researcher and initial notes related to any key themes or reactions to the research were recorded.
(ii) Notes were recorded on segments of text which were judged to contain a meaningful idea. These meaning units were used to divide the transcript.
(iii) Each meaning unit was further analyzed to understand its core idea.
(iv) Themes which represented a summary of meaningful ideas were identified for each meaning unit. While there was an inevitable level of interpretation throughout the data analysis, the team endeavored to stay close to the participants own words when locating themes.
(v) Domains were initially identified based on initial impressions of the data, a review of the literature and on the interview questions. These broad domains provided an organizing structure and a conceptual framework for the data. They facilitated the data analysis process by starting with a top down rather than a bottom up approach.
(vi) Each meaning unit and theme was then allocated to a main domain.
(vii) Themes were grouped with other themes which contained similar ideas to form categories.
(viii) Themes within categories were then reviewed and grouped with similar themes to form subcategories. The intention within this stage of the data analysis was to create an overall or abstracted meaning from the data which still reflected the underlying data at an interpretative rather than a descriptive level.

Based on the recommendation by Hill et al. (2005) that themes are labeled to provide a common unit for describing results and to further aid with future between study comparisons the following theme labels will be applied when describing the findings; Themes applying to seven or more participants are referred to as *general themes*; Themes applying to five or six participants are referred to as *typical themes*; Themes applying to two, three, and four participants are referred to as *variant themes.*

### Credibility and Trustworthiness

The credibility and trustworthiness of the data was assured through multiple processes in line with guidelines for completing and reporting qualitative research (Tong et al., 2007; Yardley, 2008). For example, all participants were sent their transcripts and asked to review them and given the opportunity to add, delete or rework any data that they felt did not accurately represent their experiences. Furthermore, several samples of the analysis were discussed and cross analyzed by the research team. Consensus was agreed on the meaning units, categories, core ideas, and themes/categories within these transcripts. Five of the interviews were fully analyzed by three researchers, two trainee psychologists and one research psychologist. Therefore, four auditors were involved in validating and modifying the analysis of the primary researcher. All data analysis was then further cross checked and closely reviewed by the research supervisor. During the write up, the primary researcher grounded Domains, Categories, and Themes/Subcategories in multiple examples from the transcripts. This was completed with the aim of illustrating examples and demonstrating a fit between the data and the meaning that was assigned.

### Ethical Approval

Ethical approval for this research study was obtained from the School of Psychology Research Ethics Committee, Trinity College Dublin.

## Results and Discussion

### The Emergence of Depression

The first domain that emerged from the thematic analysis was entitled; *The emergence of depression*. This domain which consisted of three categories and 10 themes is presented in **Table 2.**

**Table 2:** Domain 1: The emergence of depression.

Categories and Themes and No. of participants disclosing the theme
**Category 1: Extreme athletic identity and elite sporting pressures as important vulnerability factors for depression**
Exclusive identity on the all-consuming demands of sport (7/8)
Sporting performance publicly evaluated and perceived acceptance in elite (6/8) environment (from coaches/fans/family/sponsors) conditional on results
Global self-worth conditional on result and levels of perceived acceptance in elite sport (6/8)
Emphasis on and the need to hide frailties and project images of strength (6/8)
**Category 2: Intrinsic characteristics, extrinsic motivations, external locus of evaluation, and their relationship with depression**
Obsessive drive and will to win (8/8)
Playing sport to prove worth and gain acceptance from others (family/coaches) (5/8)
**Category 3: Perceived precipitating factors for depression**
Inability to cope with broader life stressors/vulnerabilities or adjust in the Offseason (6/8)
Unacceptable results or loss of skills shown in competition (5/8)
Obsessive drive and not feeling able to practice self-care in the context (3/8) of persistent financial/sponsorship demands (3/8)
Adjusting to post-competition void regardless of result and/or the anti-climax and lack of satisfaction derived from success (3/8)


While the benefits to having a salient athletic identity have been discussed in previous literature (e.g., Horton and Mack, 2000), the participants experiences in this paper reflect what Brewer et al. (1993) termed the ‘Achilles heel’ associated with having an overly salient athletic understanding of self. Central to the athletes understanding of the development of their depression were issues pertaining to identity and an ‘unhealthy’ or ‘dysfunctional’ relationship with sport. Indeed, an exclusive identity on the all-consuming demands of sport represented a general theme within the findings. One participant stated; “I was eating and drinking and sleeping sport, it was my focus every day. Whatever in my life that had to be jigged around, my mind was on it” (p. 2). The social influence on identity development (Stryker and Burke, 2000) was clearly expressed in this study as the athletes recounted how their athletic sense of self became more salient through the positive reinforcement received within the wider sporting community. Another important related vulnerability factor for depression was expressed in the theme: Sporting performance publically evaluated and perceived acceptance in elite environment conditional on results. This finding could be compared to previous research that captures the highly demanding and pressurized elite sporting environment, where performance narratives are observed to be the dominant message that athletes internalize (Carless and Douglas, 2009). One participant reflected; “A positive and negative reaction (from coaches/sponsors) is based on performance, we appreciate and affirm each other for things that we do as opposed to who we actually are” (p. 6). The typically endorsed theme; Global self-worth conditional on results and levels of perceived acceptance in elite sport further emphasizes how for most of the participants their “worth was on the line when playing sport” (p. 3). Drawing on person centered theory (Mearns et al., 2013), it could be suggested that while engaging with sport under these perceived ‘conditions of worth,’ it is understandable that the athletes typically developed an external locus of evaluation and endorsed the theme: Playing sport to prove worth and gain acceptance from others. Previous empirical research has suggested that high degrees of external motivation can have a detrimental impact on an athlete’s wellbeing and overall functioning in sport (Lemyre et al., 2007).

Another important component of the athletes understanding of depression was related to their relationship with the masculine values espoused in the elite sport context (Steinfeldt and Steinfeldt, 2010). While conformity to masculine norms is often observed to be adaptive is some contexts (Levant and Kopecky, 1995), when difficulties in their life emerged there was little space for the participants to admit despair and express vulnerability. A typical theme endorsed within this domain; Emphasize on and the need to hide frailties and project images of strength speaks to messages they received from the sporting culture and internalized as an important component of their athletic identity. One participant reflected: “In sport being tough and being driven are really admired” (p. 8). The theme further supports the argument that in addition to their worth being conditional on results, performances, and actions on the sporting stage, they were expected to express positivity, deny weakness, display emotionless qualities and fit the script of the mentally tough athlete.

A typical theme endorsed by the athletes reflected the: obsessive drive and will to win they had during their sporting careers. While this dedication, which one participant described as “bloody mindedness, not willing to stop doing something” (p. 7) is positively reinforced in sporting environments and is a characteristic that is central to being an elite level athlete (Jones et al., 2007), for some participants it was a psychological trait that when coupled with the aforementioned external demands contributed to the development or rendered them vulnerable to depression. This was observed in the variant theme: Obsessive drive and not feeling able to practice self-care in the context of persistent financial/sponsorship demands.

An important typical precipitating factor for depression in this study reflected: Unacceptable results or loss of skills shown in competition. This fits with the term ‘narrative wreckage’ recently employed by Carless and Douglas (2009) to describe the psychological impact and emotional consequences of failing to live up to the internalized performance narrative in the highest echelons of their sport. Central to the concept of identity foreclosure is the idea that it closes off any further exploration of other identities or social roles (Warriner and Lavallee, 2008). Athletes in previous studies infused all areas of life with sport while having diminished concern or time to focus on broader life concerns (Lavallee and Robinson, 2007; Carless and Douglas, 2009). Within this study, participants typically endorsed the theme; Inability to cope with broader life stressors/vulnerabilities or adjust in the off season. For some of these participants their sporting career had masked problems or had not allowed space to develop skills to deal with life outside the athletic domain. “Sport had masked problems in my personal life, on some level I had been using it to mask over my cracks and keep me going” (p. 1). On some level this fits with Miller and Kerr’s (2002) suggestion that athletes are encouraged to develop performance excellence in sport at the expense of developing a multidimensional self. A related variant theme that was endorsed reflected the precipitating factor for depression; Adjusting to post competition void regardless of result and/or the anti-climax and lack of satisfaction derived from success. While sport demanded so much attention for such prolonged periods of time, during the off season or in moments when they had space to reflect on their life and on their experiences, more uncomfortable emotions or questions about their existence began to emerge. For some of the athletes, these vulnerable experiences and existential concerns arose even after achieving their goals and childhood dreams. This finding can be compared to retirement experiences where athletes have spoken about feeling lost or undefined and not having an understanding of self when their careers came to an end (Lavallee and Robinson, 2007). These findings differ in the fact that such emotional difficulties arose during rather than post career.

### The Manifestation of Symptoms of Depression

The second domain in the findings was entitled: The manifes tation of symptoms of depression. This domain which consisted of three categories and ten themes is presented in **Table 3.**

**Table 3:** Domain 2: The manifestation of symptoms of depression.

Categories and Themes and No. of participants disclosing the theme
**Category 1: Initial manifestation of depression in training and competition**
Continued competing at an elite level without initial impact on ability to function (7/8)
Inaccurate self-understanding and insight about depression (7/8)
Gaining temporary relief and avoiding depression symptomology through participating in sport (6/8)
A lack of enjoyment derived from sport and a sense of going through the motions (4/8)
Early experience of depression temporarily spurts a push into sport orientated determination and overtraining (3/8)
**Category 2: Initial manifestation of depression outside of training and competition**
Early depression symptoms more apparent away from sporting environment (5/8)
Relationship breakdowns in sport and broader life (5/8)
**Category 3: The development of depression both in and outside sport**
Natural self-critique turns to global negative self-evaluations (7/8)
Shame and hiding depression/vulnerabilities from coaches/opponents/teammates (6/8)
Depression intensifying and the inability to keep hiding depression in competition (6/8)


The participants endorsed the variant theme: A lack of enjoyment derived from sport and a sense of going through the motions. While this retrospectively represented early signs of their slide into depression, the general theme endorsed: Continued competing at an elite level without initial impact on ability to function conveys how the athletes initially responded to their difficulties. One of the athletes reflected “I was still doing fairly well, if you look back on the record books I was still maintaining a top 30 or 40 world ranking and winning an odd tournament here and there” (p. 8). Another general theme: Gaining temporary relief and avoiding depression symptomatology through participating in sport offers insight into some of the possible functions and benefits associated with taking a more action orientated or avoidant approach to their early experiences of distress. One participant described how: “playing was the escape for me. I guess that was where I was most comfortable” (p. 5).

The findings in this domain can be compared with previous qualitative explorations of depression in men (Heifner, 1997; Brownhill et al., 2005; Chuick et al., 2009; Oliffe et al., 2010). Previous empirical research suggests that depression in men often presents in more externalized and avoidant patterns where they may over invest in work, present as anxiously attached to work performance and make conscious or unconscious efforts to hide distress form their peers (Heifner, 1997; Brownhill et al., 2005; Chuick et al., 2009; Oliffe et al., 2010). Indeed, the fact that the majority of the participants related to the theme: Early depression symptoms were more apparent away from sporting environment gives further credence to the view that the athletes depression did not present, at least at its early stage in a form that would warrant a DSM-V diagnosis (American Psychiatric Association [APA], 2013). The typically endorsed theme; Relationship breakdowns both in and outside of sport further supports the presence of more externalized symptoms that have been displayed in previous research (Oliffe et al., 2010).

The participants typically endorsed the theme: Shame and hiding depression vulnerabilities from coaches/opponent’s/teammates. One participant described his internal dialog: “I can’t tell my sponsors that this is happening because I could be a liability to them, does suicidal represent your brand?” (p. 2). The athlete’s view that depression represented the antithesis of what would be accepted in sport is understandable as desired behaviors such as; ability to goal set, being self-directed, prioritizing sport over other activities, dealing with setbacks, having unshakable confidence, superior concentration skills, and pushing through pain (Crust, 2008; MacNamara et al., 2010) do not fit with the low mood, poor motivation, irritability, and lack of concentration associated with the experience of depression (American Psychiatric Association [APA], 2013). Taking the above discussion into account it is not surprising that the variant theme: Early experience of depression temporarily spurts a push into sport orientated determination and overtraining emerged within the data. “My train of thought with depression was that I am a failure, I need to be strong, as an athlete when you make a mistake you have to bounce back, the races don’t stop” (p. 6). Furthermore, given that many of the athletes’ individual sense of self was so intertwined with sporting performance and what others thought of them, returning to the sporting arena in the name of looking for success and external feedback seems an understandable desire. Another process at play is recognized in the generally endorsed them: Inaccurate self-understanding and insight about depression. It would appear that this theme is central to the shame and confusion the athletes experienced. A related general theme: Natural self-critique turns to global negative self-evaluation further conveys the internal cognitive processes that were manifesting underneath the desperate attempts to present themselves as ‘mentally tough’ in public. Such internal dialogs and inaccurate understandings of depression is observed in previous descriptions of male depression. For example, some evidence has suggested that men who align themselves to masculine norms may have difficulty in identifying and communicating emotions and affective experience (Levant et al., 2003). The findings could be compared to previous empirical research that showed how depressive symptoms triggered self-doubt and broad concerns about having a ‘faulty’ masculinity (Oliffe et al., 2010). It could be suggested that additional factors may impact on athletes understanding of depression. For example, a number of the athletes in the study understood their early depression as a lack of mental skills or poor sport psychology. “I just thought it was my own mental lack, I thought my mental game was just weak” (p. 2). This highlights a possible risk that athletes may view their mental health through these performance narratives and in the process fail to develop the vocabulary to understand, recognize, and explain depression.

A typically endorsed theme: Depression intensifying and the ability to keep hiding depression in competition shows how the athletes failed in their bid to hide their distress and overcome their problems through avoidant and action orientated behaviors. In fact, many examples were seen where the depression or difficulties were exposed or came to the surface when playing sport. Previous empirical research has described similar intensifying, escalating, and cyclical patterns of depression in men where suppression leads to externalizing behaviors and a decisive event. For example, Brownhill et al. (2005) used the term ‘big build’ to convey the process of negative emotion (sadness and anger) intensifying through suppressive processes of coping. The participants in the study reported experiencing a ‘snap’ or a particular turning point when their distress and behavior reached ‘out of control’ or ‘unacceptable’ levels. “I couldn’t focus on what the task in hand was, and (perform skills in his sport), every (game) felt like a house of cards, like sooner or later I was going to collapse. (p. 3). Scholars have suggested that masculine norms limit social acceptance of depressive encounters, prohibit expression of typical depression symptomatology, restrict ways by which men can cope and encourage a pattern of both masking emotions and an escalating of self-destructive behaviors (Valkonen and Hänninen, 2013).

### Adaptive and Maladaptive Processes of Recovery

The third domain in the findings was concerned with the process of recovery and the associated helpful and hindering coping strategies employed by the athletes. This domain which consisted of three categories and 13 themes is presented in **Table 4.**

**Table 4:** Domain 3: Adaptive and maladaptive processes of recovery.

Categories and Themes and No. of participants disclosing the theme
**Category 1: The experience of maladaptive coping responses and the perceived barriers to recovery**
Isolating self from social support (6/8)
Using alcohol to gain temporary relief and to both avoid depression and associated emotions (5/8)
Lack of available psychological support or understanding of depression from others (4/8)
Not being listened to and the lack of collaboration in first experience of treatment (4/8)
Dealing with publicity and the continued expectations from elite sport (4/8)
Overtraining interpreted as self-harm (3/8)
**Category 2: Adaptive processes and turning points in recovery**
Separate from elite sport environment to understand depression and embark on self-discovery (6/8)
Channeling sporting will to win and personal agency toward a high level of commitment to recovery (6/8)
Experiencing acceptance and expressing real self in therapeutic relationship (5/8)
Support from significant other, recognizing depression and developing hope (4/8)
**Category 3: The process of recovery and the transitions within the self**
Being less defined by sport, broadening identity, and adopting self-care (5/8)
Developing intrinsic motivation an internal locus of evaluation and falling in love with sport again (5/8)
Coming out and gaining self-acceptance in sport and society as central to healing and recovery (4/8)


Overtraining interpreted as self-harm which was expressed as a variant theme within the data reflects how extreme overinvestment in sporting activities may have served the function of a cry for help and a maladaptive attempt to communicate their internal distress (O’Connor et al., 1989; Armstrong et al., 1991; Raglin, 1993). “I am overtraining in the gym, to the point where my hands were bleeding” (p. 4). Considering the content of earlier discussions that outlined psychological characteristics espoused and encouraged within the domain of elite sport, (for example the ability to focus and block out distractions, competitiveness, hard-work ethic, ability to set and achieve goals, pushing self to limits) it is understandable how such extreme behaviors could possibly have been reinforced or at least not recognized as problematic in nature.

A sport specific barrier to recovery was seen in the typically endorsed theme; Dealing with publicity and the continued expectations from elite sport. It would appear that the external pressures and conditions of worth that impacted on the development and initial maintenance of depression, further exerted their influence as the athletes embarked on recovery. For some of the participants they felt challenged in their attempt to bring changes in self, for example attitudes to self-care into the athletic domain. This supports previous empirical research that emphasized how athletes often felt silenced in their attempt to construct attitudes and behaviors that did not fit within the conditions of worth espoused in that pressurized and demanding environment (Carless and Douglas, 2009). A variant theme endorsed within the interviews: A lack of psychological support or understanding of depression from others reflects another barrier to recovery. The lack of understanding from others regarding depression could be linked to previous authors who have described how our tendency to idealize athletes often encourages us to not understand or observe mental health issues in those who play elite sport (Reardon and Factor, 2010).

The variant theme: Support from significant other, recognizing depression and developing hope which was endorsed by some of the participants conveys how interventions from significant others often represented the turning point in their story of depression. Indeed, social support has been established as a pertinent protective factor in the development of depression (Carr and McNulty, 2014), while an external intervention or interruption was central to the beginning of the recovery process specifically with males encountering depression (Pederson and Vogel, 2007).

Previous empirical research not directly studying depression has shown how athletes needed to gain ‘asylum’ or break away from sport in efforts to escape performance values and deal with emotional distress (Carless and Douglas, 2009). Those findings can be observed in this study within the typically endorsed them; Separate from elite sport environment to understand depression and embark on self-discovery. Considering many of the participants understood their depression as a consequence of their identity being intertwined to their performance as an athlete, and in further reflecting on the masculine dominant values that seemed to discourage emotional expression and self-reflection, it is understandable that they needed to gain distance and perspective from this environment during their attempts to understand and recover from depression. One participant reflected: “I felt I needed to find myself away from sport, find out who I was as a person” (p. 1).

While sporting characteristics were perceived as possible etiological and maintaining factors for depression, there was a clear sense within the data that many of these factors were protective in nature and used to their advantage in recovery. This is supported in the typically endorsed theme; Channeling sporting will to win and personal agency toward a high level of commitment to recovery. As one athlete reflected, ‘what got me here, got me back out again.’ (p. 4). It was observed that as they recognized they had depression, they took personal responsibility and embarked on their therapeutic journeys with the same focus and commitment that was demanded of them in their respective sports. Indeed, the personal characteristics of the clients, for example having high levels of motivation and belief in the process of therapy is viewed as central to successful outcomes (Paulson et al., 1999).

The typically endorsed theme: Experiencing acceptance and expressing real self in therapeutic relationship emerged as a decisive component to adaptive coping and supports previous empirical research that has emphasized the importance of emotional expression, receiving acceptance, and building a strong professional relationship within a therapeutic intervention (Paulson et al., 1999). Furthermore, a central aspect of the athletes understanding of their recovery is observed in the typically endorsed theme; Being less defined by sport, broadening identity and adopting self-care. In many ways this theme reflects the result of taking the time to develop what Miller and Kerr (2002) referred to as the person behind the athlete. As one athlete reflected; ‘I went back as a different person, as a different athlete, had to re-invent myself, sport wasn’t the whole me’ (p. 5). Indeed, previous research has shown that athletes can resist performance narratives or indeed adopt new narratives from which to participate in sport (Carless and Douglas, 2012, 2013).

The typical theme: Developing intrinsic motivation an internal locus of evaluations and falling in love with sport again represented another aspect of the athlete’s new sense of self. It supports previous research that emphasized the importance of having a self-defined motivation for competing in sport (Lemyre et al., 2007). Incorporating an internal locus of evaluation is observed to be central to an individual’s overall health and wellbeing (Mearns et al., 2013). “I came to a point in my life where I was able to realize that there is a lot of things that I could control, but there were things I couldn’t control in my life, other people’s reactions” (p. 7). Another aspect of their recovery is reflected in the theme: Coming out and gaining self-acceptance in sport and society as central to healing and recovery. While previous themes have reflected on the negative impact sport had on recovery, four of the athlete’s emphasized how being accepted for having had depression within their sporting careers consolidated the recovery process. The fact that they had previously often felt silenced, misunderstood or not valued for their whole being in this environment, seemed to add to the power of this acceptance.

### Strengths and Limitations

Given the challenges associated with accessing an elite sport population (Beamon, 2012) the profile of the participants and the classification of their levels of expertise into specific categories developed by Swann et al. (2015) are recognized strengths of this study. While seven out of the eight participants confirmed that they were diagnosed with depression, no third party information was sought and comorbidity which is observed as the norm with depression (American Psychiatric Association [APA], 2013), was not accounted for in this study. It is possible that the findings representing the experience of depression may be a function of some correlated comorbid disorder.

Researchers have previously called into question the reliability of employing retrospective designs. Such criticisms have largely revolved around issues pertaining to possible recall bias. This seems particularly relevant to depression as evidence suggests that memory loss is a possible side effect of MDD (Gotlib and Hammen, 2008). The current study contained two participants who reflected on depressive experiences that occurred up to and beyond 10 years prior to the interviews.

### Implications of Research

While it is beyond the scope of the study to decipher the intricacies of each participant’s experience with depression, the findings offer numerous themes that from the athlete’s perspective, provide a broad picture of some of the important aspects. While the participants, experiences can be viewed from a multitude of perspectives, including person centered theory (e.g., Mearns et al., 2013), masculine frameworks (Addis, 2008), and theories of identity (Brewer et al., 1993), the processes that underlie the athletes’ experiences can only be hypothesized.

Data that emerged across all three domains may provide elite sport coaches/teams and organizations with understanding and insight into the personal vulnerabilities that athletes may experience beneath the tough exterior that they are likely to portray. It may further draw their attention to the role that sport may have in the development and maintenance of psychological distress. With specific regard to the experience of depression, it may be important for individuals working with athletes to be aware of some of the more atypical, masked or sport specific expressions of depression. Furthermore, while mental toughness and psychological resilience is encouraged within the elite sport environment, this study would argue that these skills and attributes should not be fostered at the expense of healthy emotional expression. The findings support previous studies which have reflected on the importance of developing all aspects of the person behind the athlete and encouraging an environment where personal development and professional excellence are equalled (Miller and Kerr, 2002).

This paper may provide mental health professionals with some important insights into areas related to detection, assessment, and treatment of depression with male athletes. As recognized in previous qualitative explorations of depression in men in non-sporting contexts (Martin et al., 2013), it may be important to look beyond the DSM criteria (American Psychiatric Association [APA], 2013) as the data suggests that depression may manifest in more atypical, externalized and masked forms in this particular population. For example, the athletes in this study suggested that their outward appearance and actions (performing at a high level, training, conversing with teammates) did not match the underlying distress they were experiencing. It could be suggested that psychologists working with athletes would need to be extra prudent when assessing for depression. For example, it may be important to ask more questions pertaining to their functioning away from sport. Relationship breakdowns and over investment in training may be possible signs as depression may initially manifest through these processes. Effective psychological formulations and therapeutic interventions would likely somewhat rely upon the clinicians ability to draw on positive athlete traits while providing the core conditions so central to the humanistic therapies (Mearns et al., 2013).

### Future Research

As aforementioned, the experience of depression is seen to be heavily influenced on context (Cochran and Rabinowitz, 2000). It is worth noting that many types of sports have varying demands and differing cultural influences and expectations (Steinfeldt and Steinfeldt, 2012). While elite athletes in any sport may encounter ubiquitous challenges, future research may benefit from exploring similar lines of enquiry in one specific context, for example in elite male rugby. There is also a need to examine in more depth moderating factors (e.g., athletic sense of self and sense of identity and masculine elite sport environments) for the relationship between depression and participation in elite sport.

## Conclusion

This paper represents the first published study to explore elite male athlete’s experiences of depression during their sporting careers. The findings give insight into how the culture of sport and the interplay between the athletes sense of self and the elite performance environment influenced how they experienced, expressed, and responded to depression during their careers. The three domains represent the major themes disclosed in the course of data collection and illustrate a categorical interpretation of how the emergence of depression was recognized, the nature of how depression manifested in the participants and how the athletes navigated their way through the recovery process.

Data analysis suggested how masculine values, commitment to excellence and high levels of athletic identity which were embraced by the athletes and reinforced by the elite sporting environment played a role in the development and maintenance of their depression. The findings further point to how the male athletes experienced and responded to depression during their careers was influenced by a myriad of factors embedded in the masculine elite sport performance focused environment. The athletes performing within the elite sport culture appeared to respond to depression often with further investment in sport and with more atypical, externalizing or avoidance expressions of their internal distress. The recovery process was outlined and revealed ensuing periods or episodes of high, lows, and turning points. Recovery, as in most conditions was not a linear, straightforward process. Effective forms of coping included; expressing vulnerability and cementing genuine connections with others, taking a break from the elite sport environment and drawing on high levels of commitment (athlete characteristic) to understand depression. Building a broader sense of identity and returning to sport with greater self-knowledge, higher levels of self-acceptance and a more healthy relationship with sport represented central processes in recovery. While it may provide coaches, sporting organizations and mental health professionals with some preliminary insight into the area of depression in elite male sport, further research is needed to develop our understanding of this complex and understudied phenomenon.

## Author Contributions

Conceived and designed the study: SD and BH. Performed the study: SD. Analyzed the data: SD, BH, and MC. Contributed reagents/materials/analysis tools: SD, BH, and MC. Wrote the paper: SD, BH, and MC.

## Conflict of Interest Statement

The authors declare that the research was conducted in the absence of any commercial or financial relationships that could be construed as a potential conflict of interest.
